# Supplementary material for: Lithium enrichment in intracontinental rhyolite magmas leads to Li deposits in caldera basins
Source: Nat Commun. 2017 Aug 16;8:270. doi: 10.1038/s41467-017-00234-y (PMC5559592; doi:10.1038/s41467-017-00234-y)
Supplement: Supplementary file 1 — Supplementary Information [file 41467_2017_234_MOESM1_ESM.pdf]

File name: Supplementary Information
Description: Supplementary Figures

File name: Supplementary Data 1
Description: Descriptions of samples analyzed in this study.

File name: Supplementary Data 2
Description: SHRIMP-RG data obtained in this study.

File name: Supplementary Data 3
Description: End-member values used in isotopic mixing models.

File name: Peer Review File
Description:

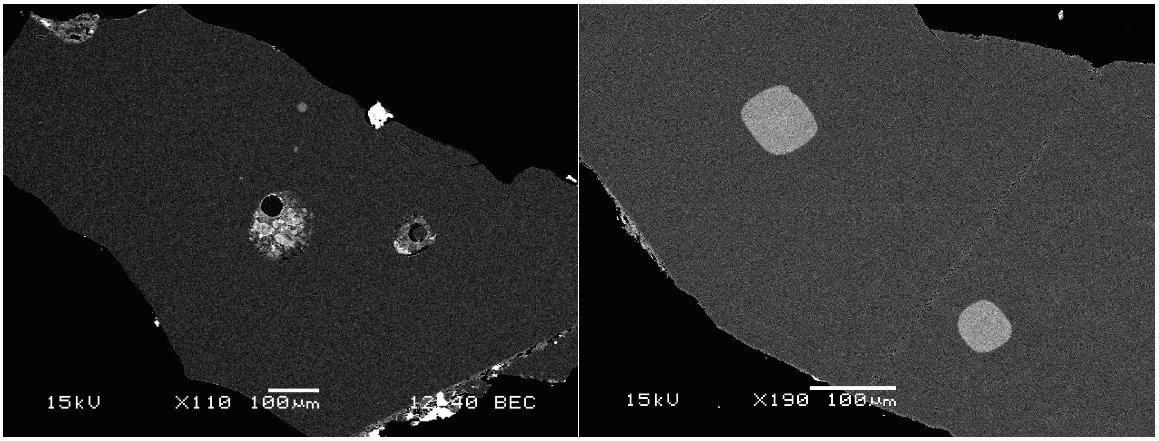

Supplementary Figure 1. Backscatter imagery of non-homogenized inclusions with vapor bubbles and crystals (left) and homogenized inclusions (right) from sample MC121, the Soldier Meadow Tuff.

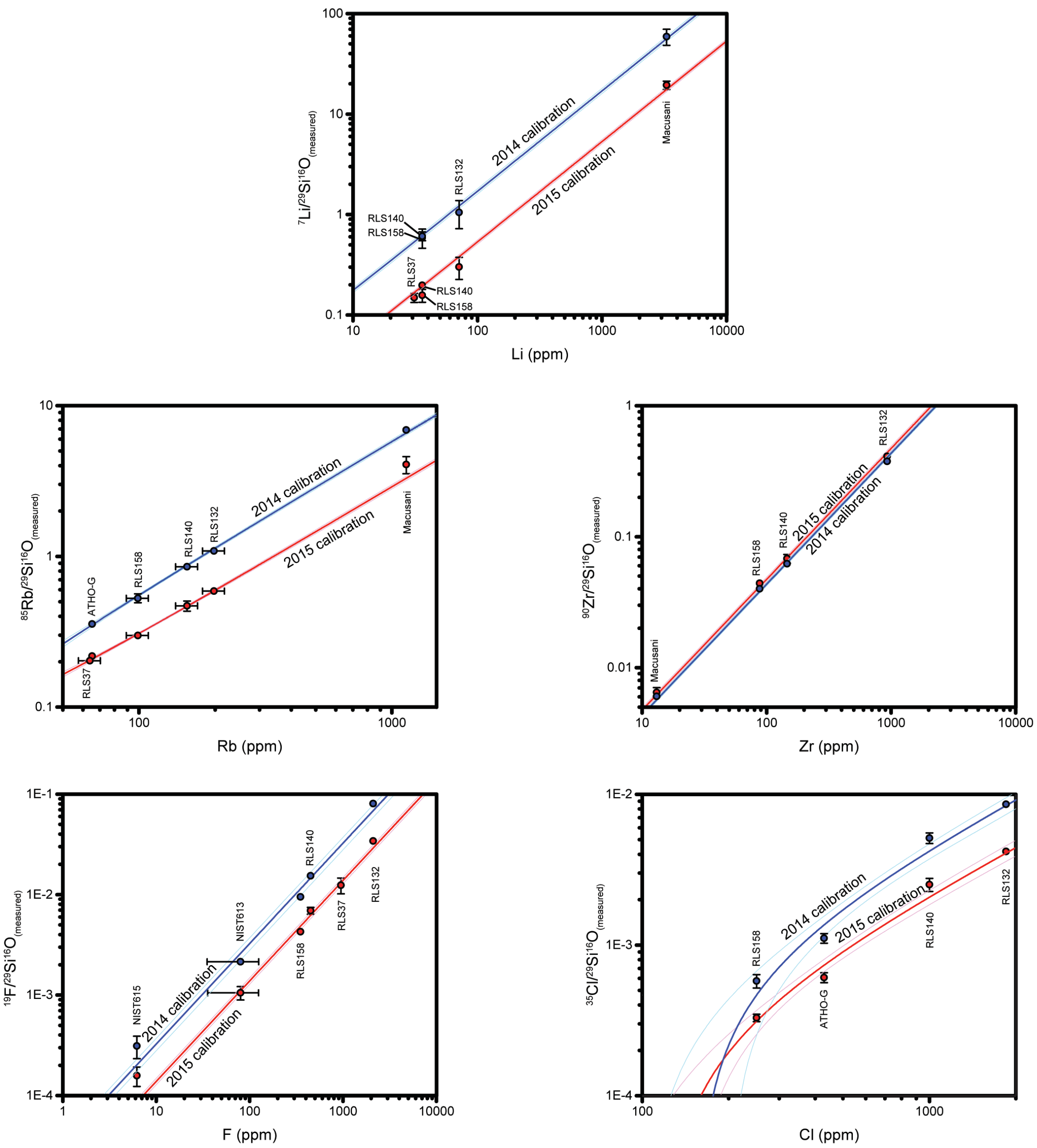

Supplementary Figure 2. Calibration curves from two different SHRIMP-RG sessions (2014, 2015) for elements discussed in the main text of the manuscript. 68% confidence bands (light colors) are used to estimate analytical error. Vertical error bars indicate SHRIMP-RG analytical errors and horizontal error bars indicate errors in standard concentrations.

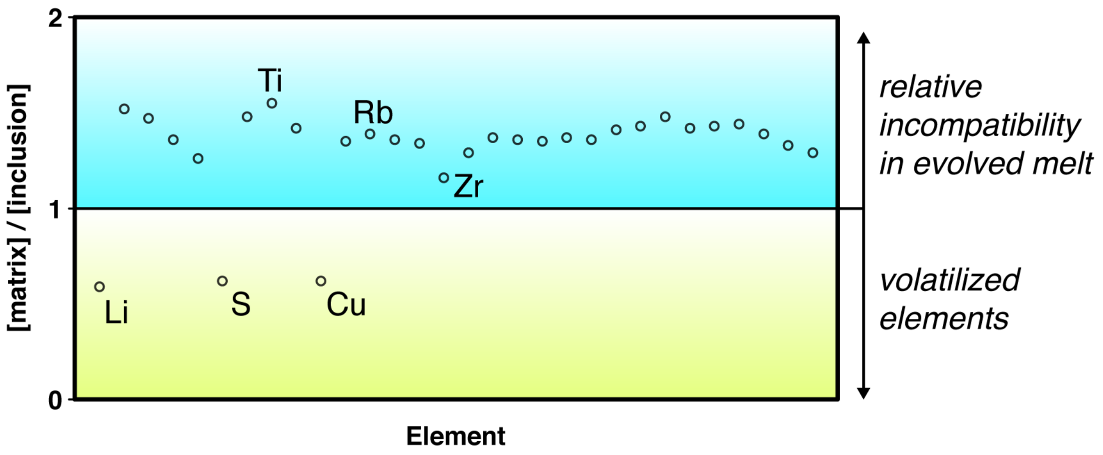

Supplementary Figure 3. Concentration of elements in matrix glass (average of 14 analyses) relative to melt inclusions (average of 7 analyses) from sample P-32, a rhyolite lava at Pantelleria, Italy. Li, S, and Cu are lost to a vapor phase after inclusion entrapment, demonstrating the need for melt inclusion analyses in any study attempting to assess the original magmatic concentrations of these elements.

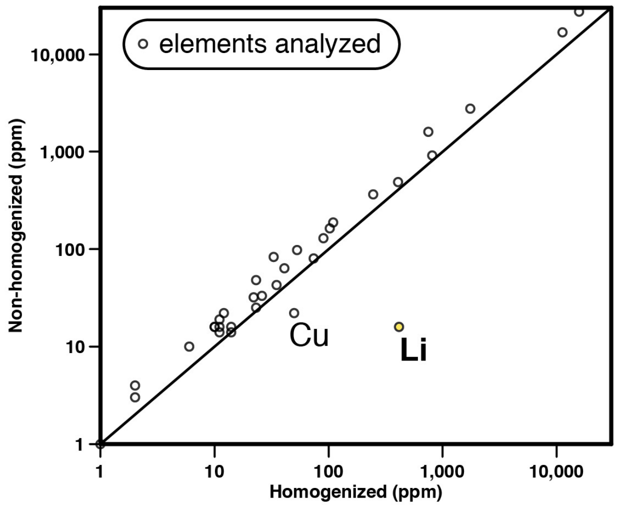

Supplementary Figure 4. Concentration of elements in homogenized and non-homogenized inclusions from the same aliquot of Soldier Meadow Tuff of the High Rock Caldera complex.

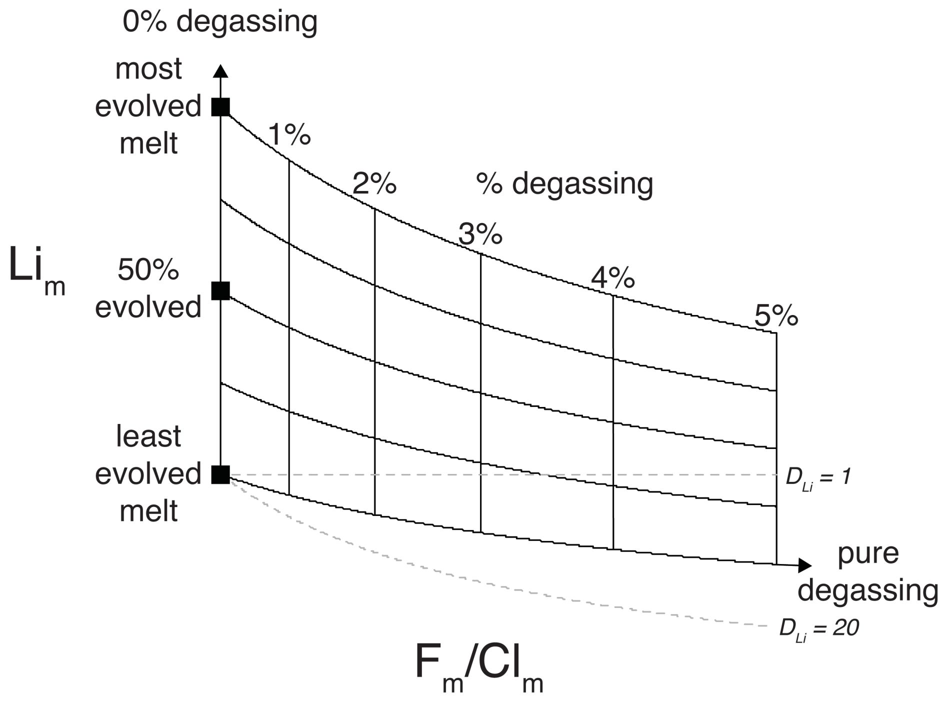

Supplementary Figure 5. Schematic representation of the interplay between magma evolution and vapor loss in analyzed melt inclusions.
